# Diverse Contexts of Zoonotic Transmission of Simian Foamy Viruses in Asia

**DOI:** 10.3201/eid1408.071430

**Published:** 2008-08

**Authors:** Lisa Jones-Engel, Cynthia C. May, Gregory A. Engel, Katherine A. Steinkraus, Michael A. Schillaci, Agustin Fuentes, Aida Rompis, Mukesh K. Chalise, Nantiya Aggimarangsee, Mohammed M. Feeroz, Richard Grant, Jonathan S. Allan, Arta Putra, I. Nengah Wandia, Robin Watanabe, LaRene Kuller, Satawat Thongsawat, Romanee Chaiwarith, Randall C. Kyes, Maxine L. Linial

**Affiliations:** * University of Washington, Seattle, Washington, USA; † Fred Hutchinson Cancer Research Center, Seattle; ‡ Swedish Hospital, Seattle; §University of Toronto, Scarborough, Toronto, Ontario, Canada; ¶ Notre Dame University, Notre Dame, Indiana, USA; # Universitas Udayana, Denpasar, Bali, Indonesia; ** Tribhuvan University, Kathmandu, Nepal; †† Chiang Mai University, Chiang Mai, Thailand; ‡‡ Jahangirnagar University, Savar, Dhaka, Bangladesh; §§ Southwest Foundation for Biomedical Research, San Antonio, Texas, USA; 1 Current affilation: SNBL USA Ltd., Everett, Washington, USA.

**Keywords:** simian foamy virus, zoonosis, macaques, Asia, retroviruses, cross-species transmission, emerging infectious diseases, primates, research

## Abstract

In Asia, contact between persons and nonhuman primates is widespread in multiple occupational and nonoccupational contexts. Simian foamy viruses (SFVs) are retroviruses that are prevalent in all species of nonhuman primates. To determine SFV prevalence in humans, we tested 305 persons who lived or worked around nonhuman primates in several South and Southeast Asian countries; 8 (2.6%) were confirmed SFV positive by Western blot and, for some, by PCR. The interspecies interactions that likely resulted in virus transmission were diverse; 5 macaque taxa were implicated as a potential source of infection. Phylogenetic analysis showed that SFV from 3 infected persons was similar to that from the nonhuman primate populations with which the infected persons reported contact. Thus, SFV infections are likely to be prevalent among persons who live or work near nonhuman primates in Asia.

## Abstract 2

These infections are likely prevalent among persons who live or work near nonhuman primates.

Human society critically influences the ecologic contexts in which the transmission of infectious agents between species occurs (*1*,*2*). In developing countries, economic growth and new infrastructure have transformed the human–animal interface, facilitating the emergence of previously unrecognized zoonotic diseases. Nowhere is this more evident than in South and Southeast Asia, where the world’s densest human populations are situated close to some of the planet’s richest reservoirs of biodiversity (*3*). Nonhuman primates figure prominently as potential sources of emerging human pathogens (*4*).

Asian cultures have long traditions of venerating nonhuman primates, and in many Asian communities nonhuman primates (particularly macaques and langurs) are woven into the fabric of everyday life (*5*–*8*). Nonoccupational interspecies contact occurs in urban settings, parks and religious sites, settings where nonhuman primates are kept as pets or performance animals, animal markets, and zoos; it also occurs during bushmeat hunting and consumption (*9*,*10*).

The first 2 contexts listed merit particular attention because they represent nonoccupational situations in which cross-species disease transmission can occur, and they represent settings in which large numbers of humans and nonhuman primates come into contact. Urban nonhuman primates are found in towns and densely populated cities throughout South and Southeast Asia, where their population may reach several thousands (*5*,*11*). This urban niche frequently and increasingly brings them into close contact with humans, as much of their food supply is provided by humans, formally or informally (as when nonhuman primates raid homes or scavenge for refuse). Temple monkeys are free-ranging in parks and religious sites in South and Southeast Asia and have lived commensally with humans for centuries at these sites, some of which have become international tourist destinations.

Simian foamy viruses (SFVs) comprise a subfamily of simian retroviruses that are ubiquitous in nonhuman primates. Ancient and well adapted, SFVs have coevolved with their nonhuman primate hosts for >30 million years (*12*). Once acquired, SFV infections are lifelong and do not seem to cause disease in their natural hosts (*13*). Nearly all captive and free-ranging macaques have acquired SFV infection by adulthood (*14*,*15*).

Studies have demonstrated that humans who are occupationally exposed to captive or free-ranging nonhuman primates can acquire SFV infection, although the number of known SFV-infected humans is small. At-risk populations include veterinarians; laboratory, temple, and zoo workers; pet owners; and bushmeat hunters (*16*–*20*). SFV prevalence in these populations is 1%–6%. The possibility of human-to-human transmission has been investigated among a small number of SFV-positive persons and their spouses and/or children. To date, no evidence of human-to-human transmission of SFV has been found (*16*,*21*).

Because of the close association of humans and nonhuman primates in Asia, most of which occurs in nonoccupational settings, we examined a large number of persons from several countries for evidence of SFV infection. All participants were asked about their past interactions with nonhuman primates, the species and population of the nonhuman primates with which they interacted, the behavioral contexts of each interaction, and the kinds of injuries, if any, inflicted. Our aim was to detect nonhuman primate–to-human SFV transmission and to learn about the behavioral contexts in which it occurs.

## Materials and Methods

### Study Sites and Populations

Our data were gathered over 7 years, and our sample totaled 305 persons (172 men, 133 women). Study sites, selected for their known human–nonhuman primate contact, were located in 4 countries in South and Southeast Asia: Thailand, Indonesia, Nepal, and Bangladesh. The seroprevalence of SFV among the nonhuman primates at these sites has been reported (*9*,*15*,*17*).

In Thailand, 211 persons were interviewed and sampled: 8 workers from a zoo in northern Thailand in 2002 and 203 persons at temples, nonhuman primate pet owners, bushmeat hunters, and urban residents from 9 sites in 2004–05. In Indonesia, biological samples and demographic and exposure data were collected from 74 temple workers at 2 sites in Bali: AK in 2000 (n = 56) and UB in 2003 (n = 18). In Nepal in 2003, 9 persons who lived and/or worked at the Swoyambhu Temple in Kathmandu were sampled; this World Heritage site is home to >400 free-ranging rhesus (*Macaca mulatta)*. In Bangladesh, where for decades ≈200 rhesus monkeys have ranged freely in the village of DH, northeast of Dhaka, 11 villagers were sampled and interviewed in 2007.

Protocols for human subject recruitment, biological sample collection, storage and handling, and collection of ethnographic/epidemiologic data have been described (*17*). Questionnaires and laboratory databases were analyzed by using NCSS 2004 (Kaysville, UT, USA) databases. Protocols for obtaining questionnaire data and biological samples were reviewed and approved by the University of Washington Human Subjects Institutional Review Board (02-5676-C06).

### SFV Assays

A bioplex whole-virus multiplex flow cytometric assay was used for SFV antibody screening. SFV was conjugated to beads as previously described for simian retrovirus, simian T-cell leukemia virus, simian immunodeficiency virus, and *Cercopithecine herpesvirus 1* (*22*). The results were validated by using plasma from known SFV-positive and SFV-negative monkeys (as determined by immunofluorescence assay). The ELISA using bacterially expressed, purified glutathione S-transferase (GST) and GST-Gag has been described (*23*). For further testing, we conducted a Western blot (WB) assay with SFV-infected or SFV-noninfected cell lysates; the SFV used was isolated from an *M. fascicularis* housed at the University of Washington. Viral bands were detected by using the TMB reagent (3,3′,5,5′-tetramethylbenzidine; Promega, Madison, WI, USA). This assay has been previously described (*15*). Each assay used a strongly positive human serum (HCM2) and negative serum sample from a person who had never been exposed to a nonhuman primate.

### Molecular and Phylogenetic Analyses

DNA was extracted from blood samples by using QIAamp Blood Mini Kits (QIAGEN, Valencia, CA, USA) according to the manufacturer’s instructions. For PCRs, the primers and conditions described by Schweizer and Neumann-Haefelin were used for *pol* (*24*), and those by Jones-Engel et al. (*17*) were used for mitochondrial sequences, with the following modifications: an annealing temperature of 52°C was used for 25 cycles in round 1 and for 29 cycles in round 2. For *gag* PCR, the following oligonucleotide primers were used: round 1 forward primer 5′-AGGATGGTGGGGACCAGCTA-3′, reverse primer 5′-GCTGCCCCTTGGTCAGAGTG-3′; round 2 forward primer 5′-CCTGGATGCAGAGCTGGATC-3′, reverse primer 5′-GAG GGAGCCTTTGTGGGATA-3′. The PCR conditions for *gag* and *pol* PCR were identical. All PCR runs included tubes containing water and noninfected human DNA as negative controls. DNA was checked for integrity by using mitochondrial DNA primers. Purified PCR fragments were cloned from round 2 into pCR2.1-TOPO by using the TOPO TA Cloning Kit for Sequencing (Invitrogen, Carlsbad, CA, USA). For each clone, 3–6 colonies were picked and purified-DNA sequenced. Sequences were analyzed by using Sequencher 4.7 (Gene Codes Corporation, Ann Arbor, MI, USA). For *pol,* 425 bp were compared; for *gag,* 1,125 bp were compared. Trimmed sequences were analyzed by using BLAST (www.ncbi.nlm.nih.gov/blast/Blast.cgi) and aligned, and neighbor-joining trees (*25*) were estimated by using the Tajima and Nei model (*26*). Bootstrap values (1,000 replicates) are represented as percentages. Positions containing gaps and missing data were not considered in the analysis. Phylogenetic analyses were conducted in MEGA (*27*). Identical results were obtained with MrBayes (*28*) (analyses not shown) under the Hasegawa, Kishino, and Yano substitution model (*28*). In those analyses a search was performed with 1 million generations, and the first 100,000 trees were discarded in the burn-in.

### Nucleotide Sequence Accession Numbers

The *gag* and *pol* gene sequences reported here were deposited in GenBank under the following accession nos.: AK04*gag* EU448349, AK04*pol* EU448363, AK19*gag* EU448350, AK19*pol* EU448364, AK23*gag* EU448351, AK23*pol* EU448365, BGH4 *gag* EU450664, HAD3 *gag* EU450665, HAD38*pol* EU448341, HAD3*pol* EU448342, MBG11*gag* EU448344, MBG13*gag* EU448345, MBG14*gag* EU448346, MBG4*gag* EU448343, MBG7*gag* EU448347, MBG8*gag* EU448348, SFVfasW*gag* EU448357, SM44*gag* EU448353, SM44*pol* EU448358, SM46*pol* EU448359, SM49*gag* EU448354, SM49*pol* EU448360, SM61*gag* EU448355, SM61*pol* EU448361, SM62*gag* EU448356, SM62*pol* EU448362, UB1*pol* EU448366, UB3*gag* EU448352, and UB3*pol* EU448367. SFVmulO is listed under accession no. DQ120937.

## Results

### Demographic Data (Table 1)

**Table 1:** Demographic and context data for 305 persons who lived and/or worked around nonhuman primates, Asia*

Characteristic	N	% Total population	% (No.) bitten	% (No.) scratched	% (No.) splashed	% (No.) SFV+
Sex						
Male	172	56.4	28.9 (50)	34.8 (60)	25.6 (45)	2.3 (4)
Female	133	43.6	28.4 (38)	28.6 (57)	23.3 (31)	3.0 (4)
Context†^*^						
Temple	234	76.7	25.6 (60)	40.2 (94)	27.4 (64)	2.1 (5)
Pet	21	6.9	52.4 (11)	42.9 (9)	38.1 (8)	9.5 (2)
Bushmeat hunting	23	7.5	0	4.3 (1)	4.3 (1)	0
Zoo work	8	2.6	75.0 (6)	100.0 (8)	0	0
Urban	19	6.2	57.9 (11)	26.3 (5)	15.8 (3)	5.3 (1)
Total	305	100.0	28.7 (88)	38.4 (117)	24.6 (75)	2.6 (8)

*SFV+, simian foamy virus positive. †Predominant form of human–nonhuman primate contact at the time of the study.

Persons ranged from 18 to 80 years of age. Their context of contact with nonhuman primates was defined as the predominant form of contact at the time of the study. Some persons reported other past contexts of contact. For example, several of the 23 bushmeat hunters, all from the same village in Thailand, had previously worked at a park where free-ranging nonhuman primates were the main attraction, and a few of the temple workers in Bali and Thailand reported having previously owned pet nonhuman primates.

### SFV Assays (Table 2)

**Table 2:** Persons at high risk for SFV, Asia*

Country	No. samples tested	No. ELISa reactive	No. WB positive	No. SFV sequences derived	Total no. confirmed SFV positive
Thailand	211†	15	3	NA	3
Nepal	9	1	1	NA	1
Indonesia	74	8	3	2	3
Bangladesh	11	1	1	1	1
Total	305	25	8	3	8 (2.6%)

*WB, Western blot; SFV, simian foamy virus; NA, not applicable. †65/213 serum samples were bioplex reactive and further screened with glutathione S-transferase-Gag ELISA.

**Figure 1:**
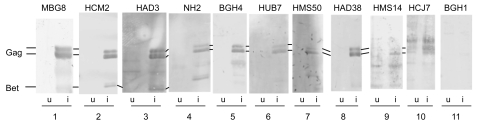
Western blot assays using human serum. Dilutions of human serum (lanes 2–11) or a foamy virus–-positive *Macaca mulatta* MBG8 (lane 1) were used to probe filter strips containing equal amounts of lysates from simian foamy virus–infected cells (from *M. fascicularis;* i lanes) or noninfected cells (u lanes). Individual strips were developed by using TMB reagent (3,3′,5,5′-tetramethylbenzidine; Promega, Madison, WI, USA). The positions of the viral proteins Gag and Bet are indicated. Lanes 10 and 11 show the range of reactivity seen with negative serum; lane 10 shows serum with nonspecific reactivity to proteins of approximately the same size as viral proteins; lane 11 shows serum negative for both lysates.

Of 305 serum samples analyzed, 211 samples from Thailand were initially screened with bioplex at the Washington National Primate Research Center (*22*), and 146 of these samples had negative results. The remaining 65 samples from Thailand and all 94 samples from Nepal, Indonesia, and Bangladesh were screened by ELISA by using GST control antigen and GST-Gag fusion protein. Of these 159 samples, reactivity of 25 exceeded GST background on ELISA, and these were further tested with WB by using SFV-infected or SFV-noninfected tissue culture cell lysates. The major reactive viral protein is the structural protein Gag. Some foamy virus–infected serum samples also react with the viral accessory protein Bet. A total of 8 (2.6%) human samples were confirmed positive by using SFV-infected tissue culture cell or noninfected cell control lysates, which all react with the Gag protein. Gag appears as a characteristic doublet of 68 and 71 kDa (Figure 1, samples 2–9). Antibody to Bet could be detected only in HCM2, HAD3, and NH2. Although reactivity of HMS14 antiserum is weak, this serum was able to neutralize SFV but not the chimpanzee-derived primate foamy virus, which confirmed infection (data not shown). All other human serum samples tested were negative for all viral proteins. Two negative examples are shown in Figure 1: HCJ7, which yielded the same background proteins in noninfected and infected lysate, and BGH1, which did not react with any proteins. Human serum samples were also tested by WB by using GST and GST-Gag protein (*15*). However, because many of the human samples reacted with GST protein, the recombinant protein WB assays were generally inconclusive (data not shown).

### Prevalence of Bites

No statistical differences in bite exposures were detected between men and women (χ^2^ = 0.009, p = 0.924, degrees of freedom = 1) or among age groups (χ^2^ = 7.678, p = 0.1043, degrees of freedom = 4). Bites were less common among bushmeat hunters (0%) and persons who lived and/or worked at monkey temples (25.6%) than among those who were exposed to urban (57.9%) and pet monkeys (52.4%). Splashes of body fluids onto mucosa were reported by nearly one fourth (24.9%) of the study population and scratches by 38.4%. Overall, 63.6% of the total population reported being exposed to nonhuman primate body fluids through a bite, scratch, or splash onto mucosa.

### Nonhuman Primate Contacts Reported by SFV-positive Persons (Table 3)

**Table 3:** Exposure characteristics of SFV-positive persons who had had contact with nonhuman primates, Asia*

Person	Sequence	Sex/age, y	Location	Context of contact	Nonhuman primate contacted	Reported exposures
HCM2	NA	M/56	Southern Thailand	Primate owner, pet	*Macaca nemestrina*	Multiple bites and scratches
HMS14	NA	F/44	Northern Thailand	Village resident, temple and pet	*M. assamensis* and *M. arctoides*	Bleeding scratches
HMS50	NA	M/43	Northern Thailand	Village resident, temple	*M. assamensis*	None
HUB7	NA	M/35	UB, Bali, Indonesia	Temple worker, temple	*M. fascicularis*	>4 bites over many y
HAD38	*gag*	F/32	AK, Bali, Indonesia	Temple worker, temple	*M. fascicularis*	2 bites within 1 y + 1 scratch
HAD3	*gagpol*	M/58	AK, Bali, Indonesia	Temple worker, primate owner, temple and pet	*M. fascicularis*	Multiple bites, scratches
NH2	NA	F/36	Kathmandu, Nepal	Village resident, temple	*M. mulatta*	Severe bite
BGH4	*gag*	F/19	DH, Bangladesh	Village resident, urban	*M. mulatta*	Severe bite 17 y ago

*SFV, Simian foamy virus; NA, not applicable. All persons were confirmed SFV positive by Western blot.

#### Thailand

At the time of sampling, HCM2, a farmer from central Thailand, was 56 years of age. Since 23 years of age, he had trained 8 pig-tailed macaques (*M. nemestrina)* to harvest coconuts. At the time of data collection, he had 3 working *M. nemestrina* that he kept in his compound and transported to the fields on his motorbike. He reported having received several scratches and 2 bleeding bites (hand and arm) over the years. The bites were treated with traditional medicines.

At the time of sampling, HMS14 was 44 years of age. She sold food at a Buddhist temple in northern Thailand and had worked and lived in the area for 30 years. Wild assamese macaques (*M. assamensis)* ranged freely through the temple grounds, commonly entered nearby homes in search of food, and frequently received food from monks and visitors to the temple. HMS14 reported that *M. assamensis* came into her home daily to raid food bins. In 1999, a pet female stump-tailed macaque (*M. arctoides)* was brought to the temple and released. HMS14 had repeated physical contact (but no bites or scratches) with this released pet macaque, which was often present at her food stall. HMS14 reported that on 3 separate occasions in 2004 she was scratched by free-ranging *M. assamensis* and that the scratches were deep enough to bleed.

HMS50, a 43-year-old laborer who had lived in a village in northern Thailand for 33 years, reported that he came to the Buddhist temple several times a week and that *M. assamensis* entered his home, near the temple, a few times a year in search of food. He reported no bites, scratches, or mucosal splashes. He did report that he fed the *M. assamensis* at the temple site.

#### Indonesia

HAD3, a 58-year-old man, worked at a Hindu temple site in central Bali, where free-ranging long-tailed macaques (*M. fascicularis*) were an attraction for domestic and international tourists. He also reported that he had previously owned 2 pet *M. fascicularis.* He reported having received >5 bleeding bites to his hands from his pet macaques and 1 bleeding bite and multiple scratches from macaques at the temple site. He did not seek medical treatment for the bites or scratches.

HAD38, a 32-year-old woman, had worked as a tourist guide at the same temple site as HAD3. She reported having received 2 bleeding bites and a bleeding scratch from the macaques within 1 year of working at this site. She applied antiseptic to her injuries.

HUB7, a 35-year-old temple worker at a Hindu temple in central Bali, reported that during his 2.5 years of work there he had been bitten 4 times by free-ranging *M. fascicularis*, once each on the hand, arm, leg, and buttock. All bites were severe enough to cause bleeding. He washed each wound with water and sought medical care, which included a tetanus vaccine and antimicrobial drugs, for the bite on his arm. He reported having been scratched only 1 time. He also had touched a pet *M. fascicularis* owned by a family in his village but had never been bitten or scratched by that macaque.

#### Nepal

NH2, a 36-year-old woman, lived immediately adjacent to the Swoyambhu Temple in Kathmandu and occasionally worked there as a cleaner. She had been bitten 1 time on her middle finger by one of the temple’s free-ranging rhesus macaques *(M. mulatta).* The wound was washed with water, and she was treated with a rabies vaccination and antimicrobial drugs at a local clinic. She denied having ever been scratched.

#### Bangladesh

BGH4, a 19-year-old housewife, was born in the central Bangladeshi village in which she was sampled. When she was 4 years old, she was severely bitten on her left calf by one of the *M. mulatta* that ranged freely through the village. She did not recall whether she had received any medical treatment. She did not report any other physical contact with nonhuman primates, though she did comment that the local macaques often entered her house in search of food, leaving urine and feces.

### Phylogenetic Analyses of SFV Sequences

**Figure 2:**
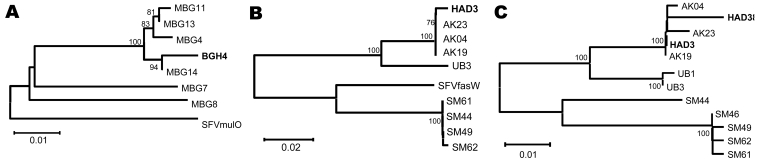
Phylogenetic trees of simian foamy virus (SFV) sequences derived from 3 persons. Human-derived SFV sequences (shown in **boldface**) were compared with those obtained from macaques of the group with which the person had been in contact and to SFV from other macaques of the same species but different geographic origin. Neighbor-joining trees A and B used gag PCR primers (1,124 bp), and C used pol PCR primers (445 bp). A) SFV gag–derived from BGH4 DNA clusters more closely (94% of bootstrap samplings) with gag sequences from 4 *Macaca mulatta* that ranged throughout her village (MBG4, MBG11, MBG13, and MBG14) than with gag sequences obtained from Bangladeshi performing monkeys, *M. mulatta* (MBG7, MBG8), of unknown origin. BGH4 gag is equidistant from gag of MBG7, MBG8, and virus obtained from SFVmulO, an *M. mulatta* of unknown origin housed at the Oregon National Regional Primate Center. B) SFV gag from HAD3, a worker at a Bali monkey temple, grouped with gag from several *M. fascicularis* (AK4, AK19, AK23) found at the same temple (100% of bootstrap samplings). UB3 is also an *M. fascicularis* Bali temple monkey that inhabited a temple ≈15 km away. HAD3-derived gag is less similar to *M. fascicularis* from Singapore (SM) and SFVfasW, an *M. fascicularis* housed at the Washington National Primate Research Center. C) Analysis of pol confirms the relationships (100% of bootstrap samplings) between SFV sequences isolated from humans (HAD3 and HAD38) and those in the corresponding nonhuman primate populations with which they reported contact (AK4, AK19, AK23). HAD3 and HAD38 worked at the same temple site where AK are found. UB1 and UB3 are *M. fascicularis* from a nearby monkey temple. Scale bars indicate number of nucleotide substitutions per site.

We derived SFV sequences from the peripheral blood DNA of 3 SFV-infected persons: BGH4, HAD3, and HAD38. We were able to amplify mitochondrial DNA from the DNA sample of another person (HCM2) from which no SFV sequences could be obtained. We have no evidence that DNA obtained from the other 4 human blood samples was of good quality (data not shown). We obtained *gag* sequences from BGH4 (Figure 2, panel **A**), *gag* and *pol* sequences from HAD3 (Figure 2, panels **B**, **C**), and *pol* sequences from HAD38 (Figure 2, panel **C**). SFV sequences from humans were compared with those from macaques of the group with which the person had been in contact and with those from other macaques of the same species but different geographic origin (Figure 2, panel **A**, *M. mulatta*; Figure 2, panels **B**, **C**, *M. fascicularis*).

SFV from BGH4 clustered most closely with SFV from 4 *M. mulatta* from her village in central Bangladesh (MBG4,11,13,14) and more distantly with 2 performing *M. mulatta* (origin unknown) sampled near her village (MBG7 and MBG8). The virus sequence of BGH4 is equidistant from that of MBG7 and MBG8 and from that obtained from an *M. mulatta* (SFVmulO of unknown origin) housed at the Oregon National Regional Primate Center. SFV *pol* and *gag* sequences from HAD3 (from central Bali) clustered most closely with SFV from AK *M. fascicularis* at the Bali temple site where HAD3 worked, as did HAD38 *pol* sequences. In contrast, the virus sequences from these 2 humans were more distantly related to those from the UB animals, which were also *M. fascicularis* but from another temple site in Bali (≈15 km away). The SFV sequences from HAD3 and HAD38 were even less similar to SFV isolated from *M. fascicularis* from Singapore (SM isolates).

These data suggest that SFV sequences are stable in nonhuman primates and can be used for several macaque species to mark an individual’s geographic origin. Correlation between the SFV sequences isolated from humans and those from the corresponding nonhuman primate populations with which they reported contact was excellent.

## Discussion

**Figure 3:**
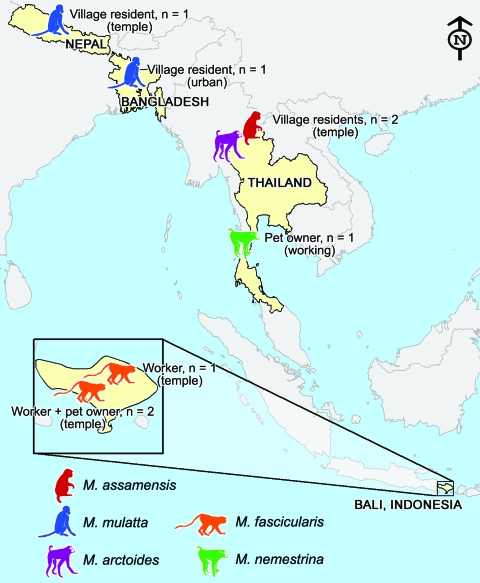
Map of the diverse contexts, countries, and nonhuman primate (*Macaca*) species associated with human infection with simian foamy virus.

We found prevalence of SFV infection in the heterogeneous populations studied to be 2.6%. In contrast with previous studies of persons who had occupational exposure to nonhuman primates, the exposure of some of the SFV-infected persons in our study was only through their normal daily routines. Previous research on nonhuman primate–human interaction in South and Southeast Asia describes interspecies contact as a frequent phenomenon in this part of the world (*29*,*30*). Our study takes this line of inquiry a step further, indicating that interspecies contact leads to nonhuman primate–to-human transmission of SFV in a variety of contexts, in several countries, and from multiple macaque species (Figure 3).

Bites from nonhuman primates are thought to be the most likely route of SFV transmission because viral RNA is found at high concentrations in the oral mucosa and saliva of infected animals (*23*). Indeed, 6 of the 8 SFV-infected persons reported having been bitten by a macaque at least 1 time. Although bites were reported by most SFV-positive persons, 2 denied having ever been bitten by a nonhuman primate. Possible explanations are that persons living in a community with a constant presence of nonhuman primates may not regard contacts, even scratches and bites, as notable events or, alternatively, that SFV is transmissible by contact other than bites, such as scratches or contact with nonhuman primate body fluids through breaks in the skin.

Other studies have shown SFV sequences to be highly stable (*12*,*31*). Switzer et al. (*19*) previously reported that they could determine the source chimpanzee of SFV infections in zoo workers by using phylogenetic analyses. We expanded those data to link SFV infections in populations exposed to free-ranging nonhuman primates to animals from their village and, in 1 instance, to differentiate native and introduced macaques solely by their SFV sequences (Figure 2, panel **A**). The 3 persons from whom SFV sequences were obtained each interacted with a single species of macaque; we did not detect any recombinant viruses, which are more likely to be encountered in persons who come into contact with multiple nonhuman primate species.

A recent review article recapitulates arguments that 2 factors influence the likelihood that disease can be transmitted from an animal reservoir to humans (*32*). First, phylogenetic relatedness suggests that the more closely a species is related to *Homo sapiens*, the more likely it is that transmission to humans can occur. The second factor is interspecies contact, which can be conceived as having 2 dimensions: the duration of contact and the intensity of contact. In general, contacts such as bites, scratches, or mucosal splashing with body fluids have the highest potential for transmitting infectious agents. In this light, the human–nonhuman primate interface in South and Southeast Asia ranks among the most likely contexts for zoonotic transmission.

In South and Southeast Asia, macaque monkeys and humans exhibit higher rates of sympatry than any other human–nonhuman primate overlap, owing in part to the major roles that nonhuman primates play in Hindu and Buddhist mythology and folklore. As a result, nonhuman primates are woven culturally and physically into the fabric of everyday life for millions of people. At least 68 temples throughout Thailand are home to populations of free-ranging nonhuman primates (*5*). Villagers in the town of Lopburi contend on a daily basis with >1,000 long-tailed macaques who spill out from the Pra Prang Sam Yot temple. These monkeys and the annual Monkey Buffet Festival (at which a buffet of fruits and vegetables is provided for all of the province’s monkeys) are a major tourist attraction. New Delhi, one of the most populous cities in the world, is also home to ≈5,000 free-ranging rhesus macaques. Interspecies contact leading to nonhuman primate bites is a familiar and increasing phenomenon in communities such as these and for their international tourists (*33*,*34*). The 5 major monkey temples in Bali collectively attract up to 700,000 visitors a year, most of whom feed monkeys and thousands of whom are bitten and/or scratched. Engel et al. recently published an analysis that used mathematical modeling to predict the likelihood of a visitor to a Balinese monkey temple becoming infected with SFV (*29*); this model predicted infection for ≈6 of every 1,000 visitors.

Two trends promise to increase human–nonhuman primate contact in South and Southeast Asia: nonhuman primate ecologic resilience and human alterations of the landscape. Because of the first trend, ecologic resilience and high birth rates, many populations of protected (sometimes fed as well) nonhuman primates are increasing rapidly. For example, during the 1990s, population levels of the 3 species of macaques in the Kowloon Hills of Hong Kong increased 100% (*35*). A second trend is habitat loss leading to increased concentrations of nonhuman primate populations in areas more densely populated by humans. In the northern Indian state of Himachal Pradesh, 86% (258,000) of rhesus macaques now inhabit urban areas as a result of habitat loss (*11*). This trend of increased urbanization of nonhuman primates is mirrored throughout Asia (*36*,*37*). In contrast, bushmeat hunting, the most common human–nonhuman primate interaction in Africa, is likely to decrease interspecies contact over time, as wild nonhuman primate populations continue to dwindle. These demographic facts lead us to echo previous calls for a global surveillance network to monitor the emergence of zoonotic disease, with the crucial caveat that such a network focus on areas of highest interspecies contact.

Our data suggest that the number of persons at risk for infection with SFV is much larger in South and Southeast Asia than elsewhere. This finding presents both a challenge and an opportunity for future research. The challenge is to find infected persons and follow the course of infection in addition to taking action to prevent future transmission. The opportunity lies in assembling a large cohort of infected persons, which will enable the use of epidemiologic techniques to learn about the natural course of SFV infection in humans.
